# Intelligent Fruit Yield Estimation for Orchards Using Deep Learning Based Semantic Segmentation Techniques—A Review

**DOI:** 10.3389/fpls.2021.684328

**Published:** 2021-06-25

**Authors:** Prabhakar Maheswari, Purushothaman Raja, Orly Enrique Apolo-Apolo, Manuel Pérez-Ruiz

**Affiliations:** ^1^School of Mechanical Engineering, SASTRA Deemed University, Thanjavur, India; ^2^Departamento de Ingeniería Aeroespacial y Mecánica de Fluidos, Área de Ingeniería Agroforestal, Universidad de Sevilla, Seville, Spain

**Keywords:** precision agriculture, yield estimation, deep learning, semantic segmentation, fruit detection and localization

## Abstract

Smart farming employs intelligent systems for every domain of agriculture to obtain sustainable economic growth with the available resources using advanced technologies. Deep Learning (DL) is a sophisticated artificial neural network architecture that provides state-of-the-art results in smart farming applications. One of the main tasks in this domain is yield estimation. Manual yield estimation undergoes many hurdles such as labor-intensive, time-consuming, imprecise results, etc. These issues motivate the development of an intelligent fruit yield estimation system that offers more benefits to the farmers in deciding harvesting, marketing, etc. Semantic segmentation combined with DL adds promising results in fruit detection and localization by performing pixel-based prediction. This paper reviews the different literature employing various techniques for fruit yield estimation using DL-based semantic segmentation architectures. It also discusses the challenging issues that occur during intelligent fruit yield estimation such as sampling, collection, annotation and data augmentation, fruit detection, and counting. Results show that the fruit yield estimation employing DL-based semantic segmentation techniques yields better performance than earlier techniques because of human cognition incorporated into the architecture. Future directions like customization of DL architecture for smart-phone applications to predict the yield, development of more comprehensive model encompassing challenging situations like occlusion, overlapping and illumination variation, etc., were also discussed.

## Introduction

Sustainable agriculture is required to fulfill the growing population’s needs by properly utilizing the available resources (Kamilaris and Prenafeta-Boldu, 2018). It can be obtained by Precision Agriculture (PA), which is supported by advanced sensing and image processing systems (Gongal et al., 2015), Artificial Intelligence (AI), etc. PA was developed in the early 1980s (Stafford, 2000). By combining modern machine vision with Deep Learning (DL) architectures, PA gains a revolutionary impact in various agricultural applications, such as crop monitoring, disease detection, and intelligent yield estimation. Among these, intelligent fruit yield estimation plays a vital role in making the final decisions regarding harvesting and fruit management.

### Limitations of Manual Yield Estimation

In most countries, fruit cultivation is practiced in a large area to fulfill the worldwide demand. Therefore, improved fruit yield estimation for large orchards is required to obtain per acre fruit production and average fruit size. It enables further activities (i.e., marketing, harvesting, stock volumes, etc.) that can be planned in an effective manner by the farmers. Traditionally, fruit yield estimation has been performed by manual counting (by agricultural scientists) and leads to low precision results, high costs, as it requires expert observation, and higher time requirements for estimation. Therefore, subsequent decision making becomes a challenging task for the farmers with manual yield estimation. Hence, there is a need for an intelligent yield estimation system that overcomes the above-mentioned problems. Recently, AI-based intelligent systems for estimating fruit yield tend to provide promising results, so the problems occurring in traditional yield estimation can thereby be avoided. Further, it allows for digital agricultural systems. Machine Learning (ML) and DL are the two important techniques used in AI systems, which produces promising results in field conditions (Gongal et al., 2015; Kamilaris and Prenafeta-Boldu, 2018).

### Fruit Yield Estimation Using ML Techniques

Before the ML era, fruit detection was done by simply capturing images from orchards and detecting the prominent features from the images such as size, shape, color, and texture of the fruit using various segmentation algorithms such as K-means, watershed, contour detection, and decision trees. ML is a subfield of AI and popularly used by many researchers, as it replaces the effort imparted by human intelligence. It works with a set of algorithms and develops a trained model for (given) input features obtained from source objects (Kamilaris et al., 2017; Liakos et al., 2018). The model is then used to test real-time data, which is not trained. In PA, ML is one of the most widely used techniques for decision making related to yield estimation, soil management, plant disease management, etc. Over the past decade, many works (Payne et al., 2013; Yamamoto et al., 2014; Dorj et al., 2017; Qureshi et al., 2017) have been done in fruit yield estimation using ML techniques because of its promising capability. Payne et al. (2013) proposed a method to segment mango fruit pixels based on the color components RGB and YCbCr with texture segmentation by identifying the adjacent pixels. The results showed a squared correlation coefficient R^2^ of 0.91 when imaging on four sides and 0.74 for one side imaging. Dorj et al. (2017) developed a method to detect and segment the citrus fruits using a watershed algorithm after converting the RGB images into an HSV color space and obtained a squared correlation coefficient R^2^ value of 0.93. Some works (Stajnko and Cmelik, 2005; Malik et al., 2016; Mehta et al., 2017) adapted the size as a criterion to identify the object boundary. Even though the results are promising, these methods do not work in challenging situations such as occlusion, overlapping, and illumination variations.

Qureshi et al. (2017) proposed a method for the precise detection of fruits by analyzing images of mango tree canopies. The authors applied two approaches: The first approach dealt with identifying fruit and non-fruit pixels by applying a set of filters on the input image. The second analyzed the boundaries of mango fruits as an ellipse rather than a circular shape. Results were compared against existing ML algorithms, i.e., K-nearest neighbors (kNN) and Support Vector Machines (SVM), and the proposed method demonstrated an F1 score of 0.68. Yasar and Akdemir (2017) developed a method for orange detection using Artificial Neural Networks (ANNs) by extracting the color features obtained from an HSV color space. The detection accuracy for the test set was 89.80%. Another work proposed by Zhao et al. (2016) detected the fruit pixels of immature green citrus using Sum of Absolute Transformed Difference (SATD) method. Finally, SVM classifier was employed to eliminate the false positives (based on textural features) and obtained the precision and recall values of 0.88 and 0.80, respectively.

### Limitations of ML Techniques

Even though ML techniques perform well in most fruit detection tasks, they show poor results while performing yield estimation over a large area. Because the techniques struggle to fit the model due to poor generalization capability (Yamamoto et al., 2014; Zhao et al., 2016). DL is a recently developed neural-network-based hierarchical technique that provides promising results in almost all sectors of agriculture (Kamilaris and Prenafeta-Boldu, 2018). Intelligent fruit yield estimation using DL is an important applications of PA, which reduces the human effort considerably, as it provides high precision results and hence improved product (i.e., fruit) management (Koirala et al., 2019).

### Fruit Yield Estimation Using DL Techniques

**FIGURE 1:**
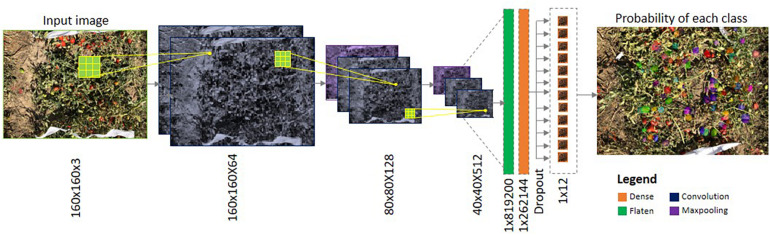
Illustration of a typical Convolutional Neural Network (CNN).

DL is a hierarchical architecture as well as a self-feature learning technique, as the layers automatically learn the features (on its own) from the raw input data (i.e., images) and hence it is more advantageous than the ML techniques. In all ML techniques, before training, the features need to be extracted from the raw input data, which is tedious and time-consuming work (Kamilaris and Prenafeta-Boldu, 2018). The Convolutional Neural Network (CNN) is one of the most widely used architectures in DL for various image recognition tasks, i.e., face recognition, tumor detection, weed detection, etc., as it is capable of handling the input image data by exploiting the spatial and temporal correlation, in a superior way. The general architecture of the CNN is shown in Figure 1. The basic CNN consists of a stack of convolutional layers, activation functions, and pooling layers. The convolutional layer is the primary layer in the CNN that performs convolution (i.e., element wise dot product) between the values of the input data and the kernel. The result is passed through the activation function in which a non-linearity operation is performed. To reduce the computational complexity, the features are then passed through the pooling layers for down sampling. Finally, at least one fully connected layer is used to provide the dense feature map, followed by the softmax layer in which, based on probability values, the image is classified into a particular class (Lecun et al., 2010). Starting with LeNet (Lecun et al., 1999), various CNN architectures were developed over the past decade, such as AlexNet (Krizhevsky et al., 2012), Visual Geometry Group16 (VGG16), VGG19 (Simonyan and Zisserman, 2015), ResNet (He et al., 2015), GoogLeNet (Szegedy et al., 2015), DenseNet (Huang et al., 2016), and SqueezeNet (Iandola et al., 2016). Each architecture differs in terms of the number of convolutional layers, non-linearity functions, type of pooling operation used, etc.

In recent years, the CNN has been used for immense applications in the agricultural sector such as disease detection and prediction of soil organic matter (Xu et al., 2019). Torres et al. (2020) reviewed the various CNN architectures, namely AlexNet, VGG16, and GoogLeNet, for fruit detection, classification, sorting, and quality control tasks. They concluded that, if the task is more complex, the kernels as well as the number of layers need to be increased for improved feature extraction. Wang and Chen (2020) proposed a method to categorize the fruits by using a deep CNN. Their architecture consists of 8 layers of CNNs and provided an overall accuracy of 95.67%. The modification in the architecture is that the authors used a non-linearity function of a parametric Rectified Linear Unit (ReLU) instead of a plain ReLU, and a dropout layer is added before each fully connected layer. It is important to note that any success of the DL architecture depends on the large amount of training data, as it is a data hungry architecture. However, collecting and labeling more training images is tedious work. Hence, Rahnemoonfar and Sheppard (2017) proposed a simulated learning method, in which training was performed using synthetic images, and testing was done on real-time data. The authors used a modified ResNet-Inception model to train the synthetic images, and the results portrayed training and testing accuracies of 93 and 91%, respectively.

### Limitations of DL Techniques

Various research studies have been performed for fruit yield estimation using DL (Wang et al., 2013; Bargoti and Underwood, 2017; Rahnemoonfar and Sheppard, 2017; Sun et al., 2018; Torres et al., 2020). In general, DL techniques perform well for most fruit detection tasks. However, one of the issues associated with the DL technique is a lower spatial resolution followed by pooling operation, which results in a poor localization of objects present in a particular scene. The exact location of the fruit needs to be localized for improved prediction. Hence, in order to obtain good accuracy in the process of automatic fruit yield estimation, DL-based semantic segmentation architectures are now employed widely (Tu et al., 2018).

This review paper is organized as follows: section “Intelligent Fruit Yield Estimation” describes fruit yield estimation employing DL-based semantic segmentation, which includes tree sampling, different sensing technologies, data augmentation methods, and different semantic segmentation architectures. The various challenges that occur when developing an intelligent fruit yield estimation system are discussed in section “Challenging Issues in Intelligent Fruit Yield Estimation System.” Section Conclusion concludes the paper.

## Intelligent Fruit Yield Estimation

**FIGURE 2:**
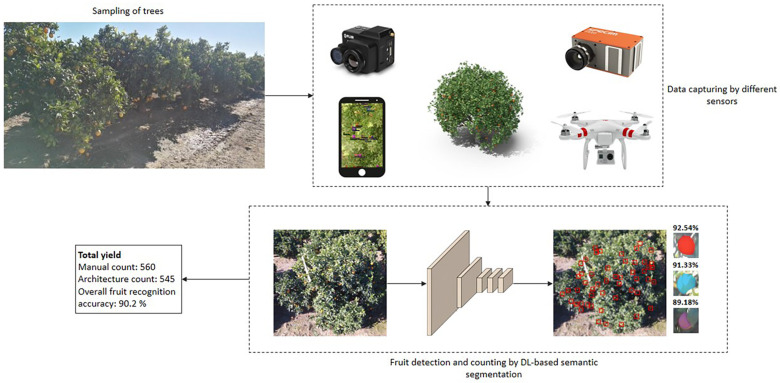
Intelligent fruit yield estimation in orchards.

The different steps involved in developing an intelligent yield estimation system are tree sampling, data capturing using different sensing technologies, data augmentation, fruit detection, counting and yield estimation using DL-based semantic segmentation architectures, and performance evaluation (as shown in Figure 2).

### Tree Sampling

Before collecting images from the orchards, the initial step is to sample the trees, which decides the number of representative trees to be taken over the entire population. The sample selection must be sufficient: it ultimately represents the whole orchard’s population, and a perfect yield can thus be estimated. Based on the survey theory, two approaches are primarily used for sampling, namely design-based and model-based approaches. For diverse populations, the design-based approach is adapted for sampling, whereas the model-based approach performs well in systems which described by the spatial positioning of a population. Various sampling procedures for design-based and model-based approaches include simple random sampling, systemic sampling, a smooth fractionator, probability proportional to size sampling, and stratified sampling, etc. (Cochran, 1977; Wulfsohn et al., 2010).

A comparative study proposed by Uribeetxebarria et al. (2018) compares the sampling efficiency in yield estimation of fruit orchards using simple random sampling and stratified sampling. The authors used the Normalized Difference Vegetation Index (NDVI) and the apparent Electrical Conductivity (ECa) for stratified sampling. As a result, the plot’s sampling size was reduced by 17% compared with simple random sampling, which improves the precision of fruit yield estimation. Table 1 shows the various sampling methods to sample the trees in an orchard.

**TABLE 1:** Various sampling methods for tree sampling in an orchard.

Sampling techniques	Description	Merits	Demerits
Simple random sampling (Cochran, 1977)	Randomly selecting samples from the whole population.	Completely represent the entire population.	Expensive and time-consuming.
Systematic random sampling (Mostafa and Ahamad, 2018)	Provides an improved tradeoff between the precision of the estimator and the sampling interval.	Faster than simple random sampling.	Realization is difficult without knowing all the members in the population.
Stratified sampling (Uribeetxebarria et al., 2018)	From the whole population, strata or sub categories are considered. Samples are taken from these strata randomly.	High precision and requires smaller samples.	If the sub category is not properly chosen, it is challenging to represent the entire population.
Smooth fractionator (Gundersen, 2002)	Systemic sampling is applied to each uniquely (based on shape, size, texture, etc.) divided unit from the whole population for efficient sampling.	Robust for the heterogeneous population.	When the population of interest is sparsely distributed, it is inefficient.
Cluster sampling (Hamilton and Hepworth, 2004)	Suitable for large and complex populations.	Minimum resources for the sampling process.	High sampling error.
Multistage sampling (Chauvet, 2015)	At various levels, sampling is performed.	More flexible.	Large number of errors due to clusters in different stages.
Probability proportional to size sampling (Gardi et al., 2008)	Each population has a size before sampling, which is proportional to the probability of selecting a unit.	Well suited for sparsely distributed populations.	Reduced precision when sampling more clustered units.

### Data Capturing Using Different Sensing Technology

**FIGURE 3:**
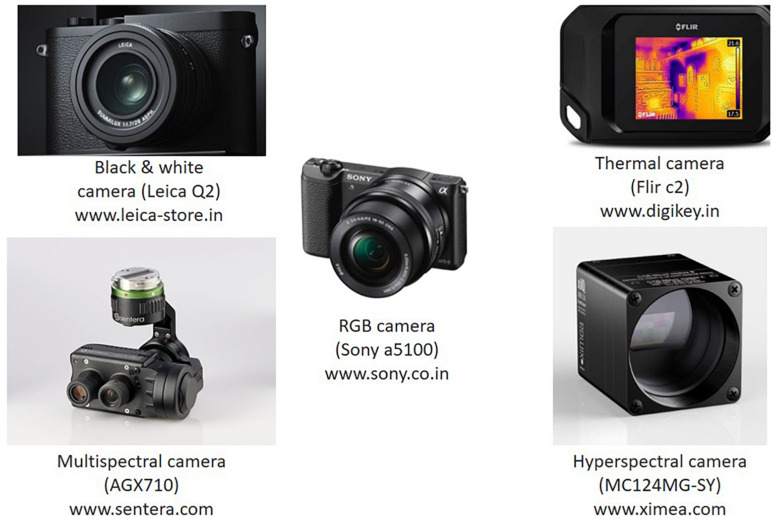
Different types of camera models using various sensor technologies.

The primary system for fruit detection is the sensing system. It should capture the focused images in field conditions by tackling challenging situations such as variable lighting conditions and resolution. Figure 3 shows the different camera models presently available in the market with various features (i.e., black and white, RGB, thermal, etc.).

Earlier studies were performed to detect fruits in orchards using black and white cameras. Without color, the widespread availability of features was exploited to detect the fruits in the canopy. After the color sensors, RGB cameras were mostly used in all detection systems (to capture the color), which made the detection process easier. Complementary Metal-Oxide-Semiconductors (CMOSs) and Charge Coupled Devices (CCDs) are prevalent technologies used as color sensors and are used widely in all machine vision systems. CCD sensors operate by capturing the entire frame simultaneously, whereas a CMOS captures the images pixel by pixel. Thermal imaging has also been used in some fruit detection works (Gongal et al., 2015). Here, each object’s feature (i.e., branch, stem, etc.) is detected based on temperature, as fruits have a higher temperature than the background objects. Spectral imaging is a next-generation camera model (i.e., multispectral and hyperspectral sensors) currently used. It gives additional information related to spectral details (at each color space) along with color features. Even if the fruit color and background (such as the leaves and stem) are the same, fruit detection can be performed using spectral information (Feng et al., 2019).

Multispectral images have fewer broader spectral bands (3–15), whereas hyperspectral images have a greater number of narrower spectral bands (20–250). The Landsat-8 satellite is an example of a multispectral imager consisting of 11 bands with a high spatial resolution of 30 m in most bands. NASA’s Airborne Visible/Infrared Imaging Spectrometer (AVIRIS) is a hyperspectral imager with 224 bands with 0.4–2.5 μm (Kwan, 2019).

Bulanon et al. (2008) explored a method for citrus fruit detection by capturing images using a thermal camera. The temperature gradient was calculated based on the emissivity of the fruit, relative humidity, and ambient temperature from the captured thermal images. Finally, these data were used to segment the fruits from the background using image processing algorithms. The results showed that the average true positive and false positive were 0.70 and 0.06, respectively.

Gan et al. (2018) developed a method for fruit detection based on multi-modal imaging i.e., combining both color and thermal images using a color thermal combined probability algorithm. It effectively extracts the information present in both images. As a result, the precision and recall rate were improved for detecting the immature green citrus fruits. Recall and precision were 78 and 86.60%, respectively, with only color images and 90.40 and 95.50%, respectively, with multimodal imaging.

Feng et al. (2019) proposed a method for detecting the apples using multispectral dynamic imaging. Using this, the pictures were taken at a considerably high level of contrast between background and fruit, which improves the recognition accuracy as 92%. Okamoto and Lee (2009) developed a method for identifying the green citrus fruit using hyperspectral imaging. The authors used the spectral wavelength of 369–1042 nm for capturing pictures and obtained a detection accuracy of 70–85%. Table 2 describes the various cameras available in the market for capturing pictures along with their resolution. The next step is the preprocessing of images, which includes data augmentation and resizing.

**TABLE 2:** Different types of camera models available on the market.

Sensors	Model	Resolution	Sensor size	References
Black and white sensor	Leica Q2	8,368 × 5,584	36 × 24 mm	www.leica-store.in
	Nikon z7	3,840 × 2,160	35.9 × 23.9 mm	www.nikon.co.in
	Canon EOS 5D	4,368 × 2,912	36 × 24 mm	www.canon-europe.com
RGB sensor	Sony a5100	6,000 × 4,000	23.5 × 15.6 mm	www.sony.co.in/
	Ricoh GR III	6,000 × 4,000	23.5 × 15.6 mm	www.ricoh-imaging.co.jp
	Fujifilm X-E3	6,000 × 4,000	23.5 × 15.6 mm	www.fujifilm-x.com
Thermal sensor	Flir c2	320 × 240	128 × 96 mm	www.digikey.in
	Testo 871	320 × 240	Not available	www.testo.com
	Fluke TI450	320 × 240	Not available	www.flukeindia.com
Multispectral sensor	AGX710	12.3 MP	89 × 88 × 98 mm	www.sentera.com
	MSC-AGRI-1-A	512 × 512	5.5 × 5.5 μm	www.spectraldevices.com
	MSC-RGBN-1-A	512 × 512	5.5 × 5.5 μm	www.spectraldevices.com
Hyperspectral sensor	MC124MG-SY	4,112 × 3,008	14.2 × 10.4 mm	www.ximea.com
	MQ022HG	2,048 × 1,088	11.3 × 6.0 mm	www.ximea.com
	OCI-UAV-1000	2048	Not available	www.bayspec.com

### Data Augmentation

**FIGURE 4:**
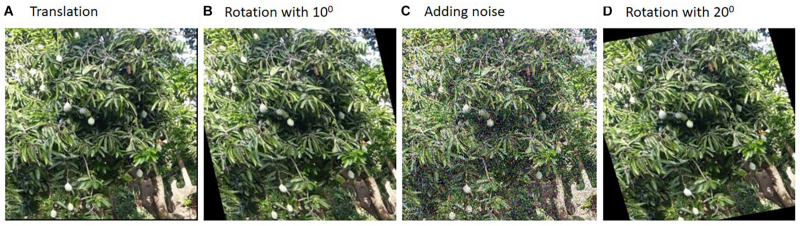
Random transformations intended for data augmentation.

Data augmentation is a useful technique in neural-network-based systems, as DL deals with a large amount of data. Practically, it is complicated to collect and annotate such a large volume of the dataset for training. However, the small dataset will lead to an over-fitting problem, and hence the system will work well in the training phase and will not produce accurate results during the testing phase. Therefore, in order to increase the available dataset to a large number, various transformations will be performed through data augmentation steps, such as translation, rotation, adding noise, cropping, and flipping. It will improve the network capability to learn the deep features present in the data (Shorten and Khoshgoftaar, 2019; Zheng et al., 2020). Figure 4 shows some of the transformations applied to a captured image for a (sample) mango tree from an orchard.

Kestur et al. (2019) proposed a method for estimating the yield of mango fruit. The authors collected 40 images with a size of 4,000 × 3,000, and it is complicated to process the same with the original size. Hence, they used the cropping augmentation method to generate the image patches with a size of 200 × 200. After augmentation, the training and testing dataset had 11,096 and 1,500 patches, respectively. The final result showed an F1 score of 0.844 for the developed architecture with data augmentation.

Stein et al. (2016), proposed a method for detecting the mango fruit using anFaster R-CNN method. In this work, the total dataset consists of 71,609 mangoes from 522 trees. They performed the augmentation by randomly sampling the subset of images from the original dense dataset, thereby capturing the entire orchard block’s variability. Image using augmentation helps to overcome the memory constraints required for processing the images. They achieved an F1 score of 0.881 in the test images.

### Implementation Using DL-Based Semantic Segmentation Techniques

For dense pixel-wise prediction, semantic segmentation is the best choice, and DL is most suitable for the hierarchical learning of data. In order to exploit its full potential, recent researchers have combined semantic segmentation with DL techniques, resulting in the highest precision accuracy, especially in the domain of fruit yield estimation. Combining semantic segmentation with DL architectures, the main objective of recent research is to obtain a perfect counting of fruits, which reveals the accurate yield for a specified orchard in challenging situations such as occlusion, overlapping and illumination variations (Payne et al., 2016; Sun et al., 2018; Guo et al., 2019).

**FIGURE 5:**
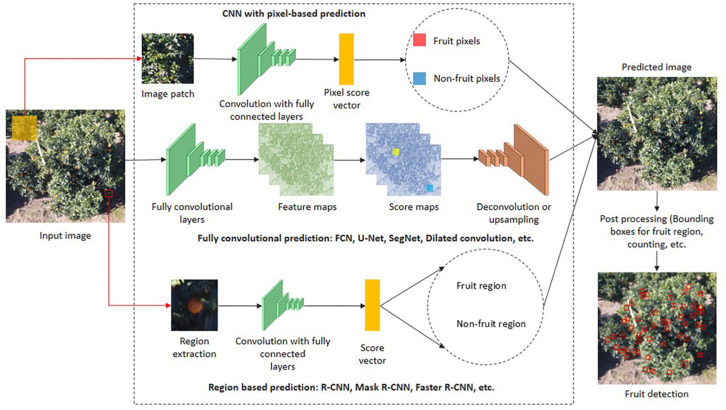
Fruit detection and localization using various DL-based semantic segmentation techniques.

Basically, fruit is detected by capturing image data and transforming it into a more detailed feature space that details every pixel present in the image. The overall DL-based semantic segmentation architectures are divided into three groups, namely a CNN with pixel-based prediction, fully convolution prediction, and region-based prediction. The first group obtains the input as an image patch and predicts each pixel into a particular class using score vectors. The second group processes the whole image, and prediction is performed based on score maps. In the third group, regions are extracted from the input image, and these regions are the input for the architectures. Based on the score vector, each region is labeled as a fruit or non-fruit region. The architectures present in each group is explained in section “Popular Semantic Segmentation Architectures in Fruit Yield Estimation.” Post-processing is then applied to the segmented pixels to group the adjacent pixels to detect the whole fruit present in a particular image. An entire process of DL-based semantic segmentation for fruit detection is depicted in Figure 5.

### Popular Semantic Segmentation Architectures in Fruit Yield Estimation

Semantic segmentation provides a complete understanding of a particular scene by labeling each pixel of an image to a specific class. It is one of the essential techniques presently used in almost such fields as agriculture, medicine, and autonomous navigation. It plays an inevitable role in object detection and localization tasks (for e.g., fruit detection in the orchards). Initially, semantic segmentation was performed using various graphical approaches such as super pixels segmentation, Markov Random Fields (MRFs), forest-based methods, Conditional Random Fields (CRFs), and dense CRFs. These methods tend to find the correlations between the adjacent pixels and obtain an inferences from them. Each pixel was labeled to a specific class based on the inference obtained from the above-mentioned graphical approaches (Shi and Malik, 2000; Ren and Malik, 2003; Silberman and Fergus, 2011; Nematollahi and Zhang, 2014; Khan et al., 2015; Yu et al., 2018).

For an improved representation of objects, the features of all objects (present in an image) need to be distinguished clearly. In this regard, hand-engineered features such as Speeded-Up Robust Features (SURF) (Woods et al., 2019), Histograms of Oriented Gradient (HOGs) (Tan et al., 2018), and Scale Invariant Feature Transform (SIFT) (Tu et al., 2018) were used to obtain reasonable features from a given image. However, in these methods, useful features need to be identified, which is a tedious process. Results of object recognition highly depend on extracted features; otherwise, the system will fail to give accurate results. For a large amount of data, the above said methods struggle to obtain complex data features.

Since the development of DL, hierarchical features have been learned from the source objects directly with hence no need for the above-mentioned hand-engineered feature learning methods. The pre-trained architectures described in section “Fruit Yield Estimation Using DL Techniques” are well suited for object detection tasks. The disadvantage is that these pre-trained architectures have suffered due to computational complexity and pooling operations. The fully connected layers present in CNNs create computational complexity, and spatial resolution has been lost due to pooling operations. Therefore, an improved localization of objects could not be achieved. Hence, DL-based semantic segmentation architectures were developed to obtain dense pixel-based prediction and improved feature learning strategies. As a result, various architectures have been explored for semantic segmentation by modifying and fine-tuning DL’s pre-trained models, namely, a CNN with pixel-based classification, FCN, SegNet, Dilated convolution, PSP Net, and weakly supervised learning models. These architectures provide perfect labeling to the raw input data and better detect all objects in a particular scene (Long et al., 2014; Badrinarayanan et al., 2017; Luo et al., 2017; Ulku and Akagunduz, 2019; Liu et al., 2019).

### CNN With Pixel-Based Prediction

In this method, image patches with a fixed size centered at each pixel are given to the CNN. In each image patch, the pixel labeling of a small region is not enough to make a localization-based decision. To overcome this issue, image patch size can be increased at the cost of more parameters calculations and hence creates computational complexity (Yu et al., 2018). This inefficient way of computation has been overcome by fully convolution prediction.

Kestur et al. (2019) proposed MangoNet, an architecture that detects and counts the fruits in an open orchard using pixel-based semantic segmentation. The original images collected from the orchard were converted into 200 × 200 image patches, and a totally 11,096 images were given to the MangoNet for training. After training, the architecture was tested with 1,500 image patches of 200 × 200 pixels from four test images. The proposed MangoNet achieved a pixel accuracy of 73.60% and an F1 score of 0.84. Table 3 shows some of the literature related to CNNs with pixel-wise prediction.

**TABLE 3:** CNN with pixel-based prediction literature.

Methodology	Authors and year	Dataset	Results
Apple detection and yield estimation using multilayered perceptron and CNN	Bargoti and Underwood, 2017	8,000 images of 1,232 × 1,616 pixel, each 32 sub images of 308 × 202 pixels obtained from each image	F1 score was 0.791, and detection F1 score was 0.861. Squared correlation coefficient R^2^ was 0.826.
Mango fruit detection and localization using multiple view geometry	Stein et al., 2016	71,609 mangoes scanned from 522 trees	Single view squared correlation coefficient R^2^ was 0.81, dual view and multi view R^2^ was ≥ 0.90.
Apple yield estimation using multi-scale sparse auto encoder feature learning method	Hung et al., 2015	8,000 apple images of the dataset Image size of 1,232 × 1,616 pixels	Squared correlation coefficient, R^2^ was 0.81. Global accuracy was 92.5%, average accuracy was 85.1%, and F1 score was 87.3%.

### Fully Convolutional Prediction

#### Fully Convolutional Networks (FCN)

**FIGURE 6:**
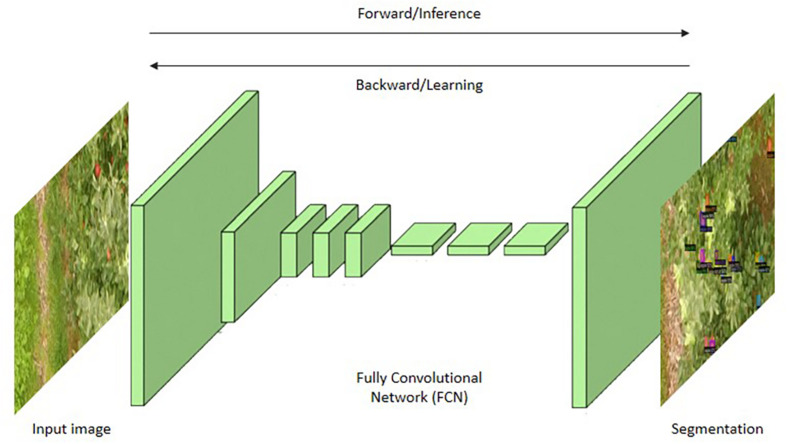
Fully Convolutional Network (FCN).

The first semantic segmentation architecture using a deep CNN was FCN (Long et al., 2014). Here, there are no fully connected layers; instead, it only has convolutional layers. The pre-trained architectures such as AlexNet, VGG16, and GoogLeNet, etc., were transformed into a new semantic segmentation architecture by fine tuning the fully connected layers. The FCN architecture is shown in Figure 6. The idea behind the FCN is that creating segmentation maps for the images of different resolutions and hence the localization of objects can be achieved by retaining the spatial resolution.

Liu et al. (2018) proposed a novel approach that uses one of the semantic segmentation FCN architectures to segment fruits from video frames. After segmentation, tracking was performed using a Hungarian algorithm with a cost function defined by a Kalman filter. Two fruit datasets (oranges and apples) with different features in the sense of variations in depth and illumination, the resemblance of color between foliage and fruit, and occlusion were used to evaluate the proposed method. An error mean of 0.20 and 3.30%, a standard deviation of 7.80 and 4.10% and a L1 error of 203 and 322 were obtained for the orange and apple datasets, respectively.

Lin et al. (2019) developed a method to detect the guava fruit using the FCN technique. The authors performed fine tuning in the original FCN architecture by employing bilinear interpolation and Adam optimizer with a learning rate of 0.0001. The trained FCN architecture was used to detect the guava fruit in the field. The proposed method’s detection accuracy in terms of precision and recall was 0.983 and 0.948, respectively.

#### Encoder-Decoder Architecture

In addition to the FCN, the encoder-decoder architecture was introduced. SegNet is one of the most popular encoder-decoder architectures that implements transposed convolution.

**FIGURE 7:**
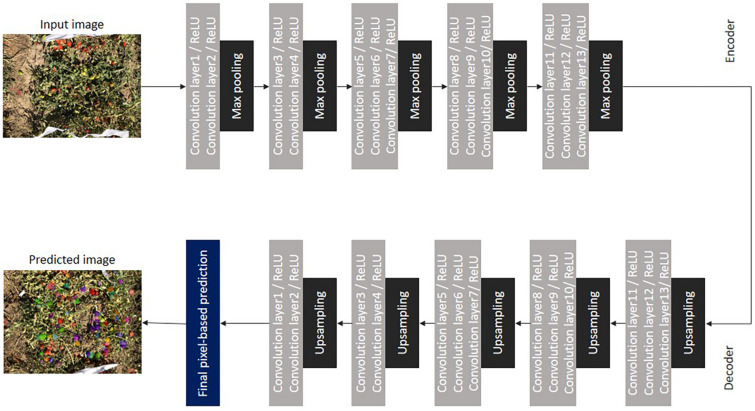
Encoder-decoder architecture of SegNet.

The SegNet architecture is shown in Figure 7. For developing this network, VGG16 was modified by removing the (last) three fully connected layers and used as an encoder. The decoder consists of 13 convolutional layers in addition to upsampling layers. The purpose of upsampling layers is to obtain indices of pooling layers. Bilinear interpolation was used in the upsampling layers for obtaining the lost size of the input due to the down sampling process (Badrinarayanan et al., 2017).

Architectures similar to SegNet were developed, namely the Deconvolution Network (DeconvNet) (Noh et al., 2015) and U-Net (Ronneberger et al., 2015). DeconvNet performs semantic segmentation at the cost of high computational resources and is very hard to train, as it has more parameters in the feature maps. U-Net differs from SegNet in upsampling operations. U-Net is designed to send the entire feature map to the decoder instead of sending the pooling indices as in SegNet, thus consuming more memory.

Hani et al. (2019) proposed a comparative study for fruit detection and yield mapping using three architectures: U-Net, the Gaussian Mixture Model (GMM), and the faster R-CNN. In the case of U-Net, 103 images of a 1,920 × 1,080 pixel size of apple trees with different varieties, tree shapes, and growing stages were used for training. The U-Net performed best and produced high recall for the given dataset. The results for fruit detection using U-Net implied that it achieves good performance when the testing dataset is similar to the training dataset, as there were poor generalization results for different testing set.

#### Dilated Convolution and Pyramid Scene Parsing Network

Another version of semantic segmentation architecture is dilated convolution (Chen et al., 2017), and it is also known as atrous-convolution or holes’ algorithm (Holschneider et al., 1989). Its operation is simply based on an undecimated wavelet transform. Using atrous-convolution by changing the receptive field’s dilation factor (Luo et al., 2017), the dense prediction was obtained in the dilated convolution. In standard convolution, the dilation rate (r) is one. By inserting zeros, the kernel size is effectively increased (with only the same non-zero values) without affecting the computation cost. Hence, the spatial resolution of the feature maps can be maintained for dense segmentation tasks.

In a deep CNN, the context information is determined by the size of the receptive field. Ongoing through high layers, the receptive field is smaller, so the network could not incorporate the global context information. Zhao et al. (2017) addressed this problem by introducing an effective global prior representation. For obtaining discriminative features among various objects present in a particular scene, this work introduced a representative method to fuse information among different sub-regions (with these receptive fields). The pyramid scene parsing technique used the ResNet architecture with a dilated network for an effective representation of the global context present in an image.

Kang and Chen (2019) proposed a backbone network to detect and segment the apples and branches in an orchard using visual sensors attached with the robotic arm. This network used the atrous pyramid spatial pooling and Gate Feature Pyramid Network (GFPN) to improve the learning capacity, at all levels of spatial resolutions. The backbone network was based on the ResNet101 architecture, which has residual connections that extract the depth features and avoid vanishing gradient problems while passing through the back propagation stage. Three models have been developed, namely DasNet-A, DaSNet-B, and DaSNet-C. The GFPN admits only the selective features as a representative (among different levels). It reduces the spatial shift, and gradient values can be balanced during the steps of back propagation.

#### Multipath Refinement Network

The Refinement Network (RefineNet) (Lin et al., 2017) is a multi-path networks that generates a high-resolution feature maps by obtaining all informations from the down sampling process. It uses residual connections in order to obtain a high-level semantic feature map, which ensures an improved segmentation of objects. The pre-trained architectures ResNet developed by He et al. (2015) was used to build refinement networks. In this regard, the ResNet is divided into four blocks, and each RefineNet block is connected to the output of the respective ResNet block. In order to obtain improved results, the developed multipath network accepts input from the multiple ResNet blocks. To the best of our knowledge, no work has yet been carried out using RefineNet, in the domain of fruit yield estimation.

### Region Based-Prediction

#### Regions With Convolutional Neural Networks (R-CNN)

In the R-CNN developed by Girshick et al. (2014) the bottom-up regions are first extracted (from the input images), and these regions are the input to the CNN for extracting the features. Finally, linear SVMs are used to classify the pixels into a particular class, and improved detection and localization can thereby be achieved for semantic segmentation. More computation time is one of the bottlenecks of the R-CNN. Hence, researchers have explored superior methods of detecting the object, namely the faster R-CNN (Ren et al., 2016). This network has a Region Proposal Network (RPN), which consists of fully convolutional layers and provides region proposals from the input images by predicting the boundaries of an object. Object detection using the R-CNN will be performed. The extension of faster R-CNN is mask R-CNN (He et al., 2017), which has an additional unit to predict the mask of an object along with the existing bound box recognition unit. The mask R-CNN is best suited for advanced stages of segmentation, i.e., instance segmentation, where each object present in an image is detected and differentiated separately by predicting the mask (for each distinct object).

Small (passion) fruit detection and counting was performed by Tu et al. (2020) using a multiple-scale faster R-CNN using RGB-Depth images. Two modules were used: the RPN and the faster R-CNN. The first module was used to generate the object proposals. These object proposals were fed as inputs to the second module, which detected the fruits with bounding boxes.

Wan and Goudos (2020) proposed a method for multi-class fruit detection using an improved faster R-CNN architecture. The authors used three varieties of fruits: apple, mango, and orange. The dataset consists of 820 apple images, 822 mango images, and 799 orange images of a 100 × 100 pixel size. The penalty factor and iteration were chosen as 200 and 5,000, respectively. The improved faster R-CNN obtained precision values of 92.51, 88.94, and 90.73% for apple, mango, and orange fruits, respectively. The processing speed was 50 ms/image. Compared with other DL-based semantic segmentation architectures, such as YOLO, fast R-CNN, faster R-CNN, YOLOv2, and YOLOv3, and the proposed method outperformed than the other architectures both in precision and processing speed.

Apolo-Apolo et al. (2020) developed a model for citrus fruit detection using faster R-CNN architecture. The images from 20 sample trees were captured from the citrus orchard using a Unmanned Aerial Vehicle (UAV). Faster R-CNN is one of the pre-trained semantic segmentation architectures used for training the orange dataset. Features extracted from the images using CNN were given as input to the region proposal network. This network consists of stack of convolutional layers followed by non-linearity function and max pooling layers. Object proposals were identified using the CNN based on the object score obtained at each position. Then, the classification, bounding box prediction and size of the objects were estimated. The proposed model achieved more than 90% precision and an F1 score of more than 89%. False positives were observed in challenging situations such as sunlight variation and immature fruits. The standard error for fruit count using the proposed model was 6.59% against the visual count. Based on the count, yield of the whole orchard was estimated using the Long Short-Term Memory (LSTM) model. It is a recurrent neural network that predicts the present data using the information obtained from the past data which helps to identify the complex pattern of the data. The real yield data collected from the orchard were compared against the estimated yield. It portrayed the standard error of 4.53%.

#### Single Shot Detectors

YOLO (You Only Look Once) refers to the detection of objects in a single pipeline, and end-to-end training is performed using a single shot detector architecture (Redmon et al., 2016). Object detection is formulated as a single regression problem by placing the bounding box coordinates to image pixels and then assigning class probabilities. The input image is divided into an m x m grid, and the specific object is detected if the center of the object comes into the grid cell. The bounding box and the confidence score are then predicted by each grid cell. There are five predictions made by each bounding box, such as the center of the box coordinates represented by the two initial predictions (i.e., x, y), the height and width related to the whole image. Final, i.e., fifth, prediction is the confidence score defined by the ratio of the Intersection over Union (IoU) value of the predicted box to the ground truth box. The model is trained with loss function, so performance can be improved significantly over the other models. Recent works pertaining to the R-CNN and single shot detectors are depicted in Table 4.

**TABLE 4:** Literature studies for fruit yield estimation using the R-CNN and single shot detectors.

Work	Authors and year	Dataset	Results
Citrus fruit yield and size estimation using faster RCNN	Apolo-Apolo et al., 2020	Images taken from (sample) 20 trees of citrus grove using a UAV during 3 consecutive campaigns	Standard error of 13.74 and 7.22% by manual and processed model predictions, respectively.
Orange fruit detection using faster mask R- CNN	Ganesh et al., 2019	Original image size was 2,816 × 1,880. Sub images of 150 were obtained with a pixel size of 256 × 256 for training. RGB and HSV multimodal data were used.	For RGB images, F1 score and precision were 0.88 and 0.89, respectively. For the mixture of RGB and HSV images, F1 score and precision were 0.88 and 0.97, respectively.
Apple fruit detection and counting using U-Net, GMM, and faster R-CNN	Hani et al., 2019	103 images of 1920 × 1080 pixel size	Overall accuracy using different architectures lies between 95.56 and 97.83%.
Citrus fruit detection using mask R-CNN	Kim and Lee, 2018	200 images of 800 × 800 pixel size	Detection accuracy was 97%
Kiwifruit detection using faster R-CNN with Zeiler and Fergus Network (ZFNet)	Fu et al., 2018	Training phase: 700 field images captured with a 2352 × 1568 pixel size. 2100 sub-images with784 × 784 pixel size Testing phase: 100 field images	Average precision during training was 89.3%. Occluded fruit was 82.5%. Overlapping fruit was 85.6%. Adjacent fruit was 94.3%. Separated fruit was 96.7%. Overall recognition ratio was 92.3%.
Grape detection using mask R-CNN, YOLOv2 and YOLOv3	Santos et al., 2020	300 images with 4,432 boxed clusters and 2,020 masked clusters	F1 score of test set was 0.889, precision was 0.92, and recall was 0.86.
Apple and pear fruit detection using modified YOLOv2	Bresilla et al., 2019	Original images: Apple: 5,000 images Augmented images: 20,000	F1 score before and after augmentation was 0.79 and 0.90, respectively.
Mango fruit load estimation using MangoYOLO	Wang et al., 2019	Two sets of video (with low and high frames) were taken to assess the performance of MangoYOLO architecture. First test set: 110 frames and second test set is 1162 frames	R^2^ values of 0.665 and 0.988 were achieved for the first and second test set, respectively.
Apple, almond and mango detection using faster R-CNN	Bargoti and Underwood, 2016	Training images: Apple: 729, Almond: 385 and Mango: 1,154. Testing images: Apple: 112, Almond: 100 and Mango: 270.	F1 score: Apple: 0.904. Almond: 0.775. Mango: 0.908.

### Weakly and Semi-Supervised Methods

Though the above feature-based scene labeling methods perform well, the main drawback is that they require more time and complex for annotation of images. This problem was explored using weakly supervised methods, and bounding box annotation was used. Multiple Instance Learning (MIL) methods presently explored new ways of learning class models. In this method, the labels in training classes are a set of positive bags rather than isolated patterns. There are (mainly) two steps in this algorithm. First, it assigns labels to all pixels in the positive bags, and learning is performed using a Support Vector Machine (SVM). In the second step, based on the SVM’s learning, it reassigns the labels to the pixels (Demiriz and Bennelt, 2001). This method (weakly and semi-supervised methods) was formulated using variant Expectation-Maximization (EM) algorithms. Based on the current estimates of the parameters and conditions provided in the observations in the expectation step, the new estimate is calculated based on the expected value under the maximum likelihood condition (Moon, 1996).

Bellocchio et al. (2019) proposed a method to count the fruits using a weakly supervised method. The authors used a new approach that employs a simple binary classifier to detect the fruits present in an image without the use of any supervision. Most of the semantic segmentation architecture used for object detection and localization discussed so far requires ground truth images, which require a more manual intervention, and this can be avoided by the proposed method. The architecture was tested with three different fruit datasets (apples, olives, and almonds), and the results were compared with the various supervision-based architectures. The authors concluded that the proposed weakly supervised architecture provided promising results equal to supervision techniques without any prior information such as ground truth labeling or bounding box information.

### Performance Evaluation

Usually, the segmented output is evaluated for its performance by comparing the results with ground truth images in semantic segmentation architectures. The widely used performance metrics are RMSE, squared correlation coefficient R^2^, pixel accuracy, recall, precision, F1 score, and IoU. The effectiveness of these measures depend on the number of pixels classified as true positive, true negative, false positive, and false negative (Yu et al., 2018).

Ganesh et al. (2019) proposed a method to detect and segment the oranges in an orchard. They measured the performance of their proposed method by using the precision, recall, and F1 score. Multi-modal input data, i.e., images taken with three different color spaces, namely, RGB, HSV, and combined RGB and HSV, were used. Among these three different (input) color spaces, the highest F1 score of 0.88, the highest precision of 0.97, and the lowest recall value of 0.60 were obtained in the combined RGB and HSV color space. Poor results were obtained with the HSV color space, as many false positives were detected.

Guava fruit segmentation using FCN architecture by Lin et al. (2019) showed a mean accuracy of 0.893 and an IoU of 0.806. The results were compared with SegNet and CART (Classification and Regression Trees Classifier) architectures, and the FCN well outperformed the other two methods. In this method, true positive and false positives values were 255 and 4, respectively, for the 91 test images. The precision and recall of the entire architecture were 0.983 and 0.949, respectively. Some of the false prediction by the proposed method was obtained due to overlapping and illumination variations. Table 5 shows the widely used performance metrics for measuring the effectiveness of semantic segmentation architectures.

**TABLE 5:** Performance metrics used for evaluating semantic segmentation architectures.

Performance metric	Description	Formulae
Root Mean Squared Error (RMSE)	Measures the squared difference between the actual output and predicted output	$RMSE=\frac{1}{m}\sum_{i=1}^{m}\left(A_{i}-P_{i}\right)$ ^2^ where*Ai* is the actual output; *P*_*i*_ is the predicted output; *m* is the number of observations.
Squared correlation coefficient (R^2^)	Measures the squared value of the linear relationship between two variables.	$R^{2}=1-\frac{MSE}{var\left(x\right)}$ where *MSE* is the Mean Square Error; *var*(*x*) is the variance of response variable *x*.
Pixel Accuracy (P*Accuracy*)	Measures the number of pixels classified correctly in each class	$P_{Accuracy}=\frac{\sum_{k=1}^{M}\delta \left(p_{k},g_{k}\right)}{M}$, where *M* is the total number of pixels present in the test images; δ(*p*_*k*_,*g*_*k*_) is the decision maker which is defined by δ(*p*_*k*_, *g*_*k*_) = $\left[\begin{array}{c} 1,{\mathrm{if p}}_{k}=g_{k}\\ 0,\mathrm{otherwise} \end{array}\right]$
Precision (P)	Corresponds to the accurate detection of fruits	$P=\frac{\sum_{k=1}^{M}\left(\delta \left(p_{k},n\right)\&\delta \left(g_{k},n\right)\right)}{\sum_{k=1}^{M}\delta \left(g_{k},n\right)}$ where *n* is the number of classes for N classes; *p*_*k*_ is the total pixels present in the predicted output; *g*_*k*_ is the total pixels present in the ground truth.
Recall (R)	The architecture efficiency is usually measured by the metric of recall	$R=\frac{\sum_{k=1}^{M}\left(\delta \left(p_{k},n\right)\&\delta \left(g_{k},n\right)\right)}{\sum_{k=1}^{M}\delta \left(p_{k},n\right)}$
F1 score (F1)	The entire fruit detection performance is indicated by the F1 score, which gives the harmonic mean value of precision and recall.	$F1=2\left(\frac{PR}{P+R}\right)$ where *P* is Precision; *R* is Recall.
Intersection over Union (IoU)	Measures the ratio between the intersection and union of the ground truth pixels and the predicted pixels of the segmented output for each class of the image	$IoU=\frac{GT_{p}⋂P_{p}}{GT_{p}⋃P_{p}}$ where*GT*_*p*_ is the ground truth pixels of each class; *P*_*p*_ is the predicted pixels of each class.

## Challenging Issues in Intelligent Fruit Yield Estimation System

### Sampling

**FIGURE 8:**
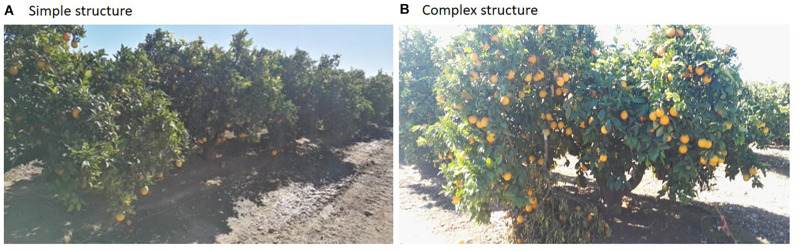
Simple and complex structures of mango orchard.

Tree sampling is the primary step in fruit yield estimation. Various sampling techniques as described in section “Tree Sampling” are available for selecting the number of trees (to sample) among the population. In the orchards, various structures of trees are present, i.e., ranging from simple to complex structures, as shown in Figure 8. Simple random sampling is unsuitable for complex structures, whereas it is sufficient for simple structures. If an orchard is segregated into clusters, it is easy to obtain a field attribute for an improved sampling strategy based on correlation. In the case of a mango orchard, trees with more branch terminals have the potential to yield a higher crop load, as it produces inflorescence at the branch terminals. Hence, an appropriate sampling strategy to choose the number of trees in an orchard needs to consider all the factors, so the precise results can be obtained with the developed intelligent yield estimation system (Wulfsohn et al., 2012).

Wulfsohn et al. (2012) developed an unbiased yield prediction estimator by evaluating a three-level systematic sampling methods. The sampling was done on 14 commercial orchards of different fruits, i.e., kiwifruit, apples, and table grapes, using the unbiased estimator with three-stage sampling units. The results showed that successful sampling was achieved with an error rate less than 5% in six orchards and with an error rate of less than 5–10% in five orchards, and the remaining three orchards deviated from the error rate by 13–20%. Sampling time for each fruit differed, taking 85–150 min for the kiwifruit, 10–100 min for the apples, and 85 min for the table grapes. Large number of trees for sampling leads to high costs in terms of time and money, and small number leads to a lower precision. Hence, sampling time is one of a critical factor that needs to be considered while taking samples from the whole population.

Various parameters, such as the objective of the work, the required precision, the heterogeneity present in the population, and the entire population size, needs to be focused while applying sampling techniques for fruit yield estimation. If said factors are well defined and based on the chosen sampling technique, fruit yield estimation can be accurate (Sharma, 2017).

### Data Collection and Annotation

**FIGURE 9:**
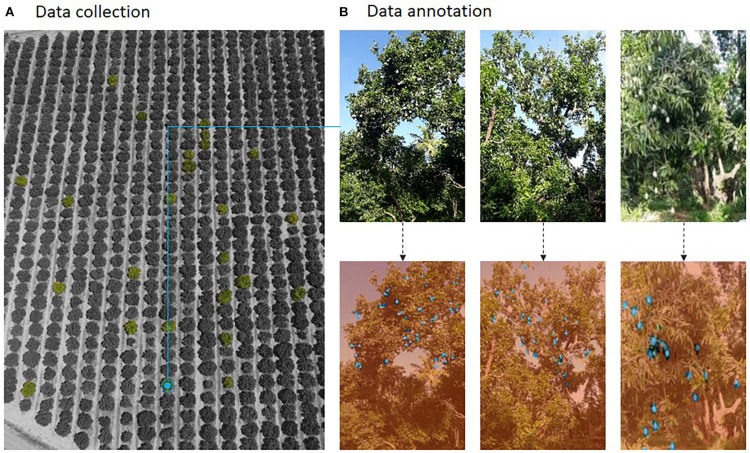
Data collection and annotation.

Collecting data from the fruit orchards and annotating are the major challenges in developing a fruit yield estimation system. Data collection should be appropriate to train the network, as it decides the learning capability of the system. Different sensing technology as discussed in section “Data Capturing Using Different Sensing Technology” provides an improved method of capturing tree images in single, double, and multiple views. Factors such as natural illumination and field of view when capturing images in an orchard will cause different effects for various sensors. Figure 9 shows a collection of images of a mango orchard and the corresponding image labeling.

After collecting images, image annotation (i.e., labeling) is to be done. Both image datasets i.e., original and annotated, are given as an input to the architecture for training. It is important to note that the manual annotation of images is tedious work in the development of an intelligent yield estimation system, as each pixel must be labeled as a fruit or non-fruit pixels. To overcome this issue, now-a-days various well established annotation tools are now available to label images in an efficient way, namely LabelImg, Labelbox, VGG image annotator, and Appen^1, 2, 3, 4^.

### Data Augmentation

Another important domains in fruit yield estimation is data augmentation. If a dataset with a smaller size is fed to the architecture, it leads to overfitting. Hence, the system is not suited for real-time applications, as it gives poor (precision) results, during the test set. Data augmentation overcomes this issue by employing various transformation techniques, as described in section “Data Augmentation.” It has to be done very carefully, as it has to deal with different concerns such as view point, occlusion, lighting, and background. These invariances need to be considered while performing transformation techniques, in order to increase the dataset. In image recognition tasks, one of the issues is class imbalance, due to which the architecture is biased toward the majority class type. This has to be overcome by oversampling during the augmentation technique. Initially, random oversampling is used, where images from the minority class type are duplicated using a naïve approach until the imbalance disappears. Followed by this traditional method, other techniques, such as synthetic minority oversampling technique and borderline synthetic minority oversampling techniques, are used to obtain improved normalized results (Shorten and Khoshgoftaar, 2019).

### Fruit Detection and Counting

#### Occlusion, Overlapping and Illumination Variation

While developing an intelligent fruit detection system, challenges such as illumination variations, occlusion, and overlapping need to be addressed, as they will cause poor recognition results. Some problems were addressed using image processing and ML techniques by Payne et al. (2016), in fruit detection and localization tasks. Results showed that detection can be improved by reducing the shadowing effect utilizing overcast and/or night time imaging. Using enhanced DL architectures, localization accuracy can be improved by detecting occluded fruit if there is a hint in an image. In big orchards, it is complicated to address all the issues, as above systems of image processing and ML techniques detect fruit based only on the color, shape, and size. Color features may not work well in various lighting conditions, and texture-based features are not enough to recognize the number of fruits in overlapping conditions. These issues may result in false predictions. Hence, a reliable system with the close cognition of human is required for improved object detection. To some extent, DL-based system is preferred for near optimal prediction, as it is a self-learning architecture.

In this context, a method was proposed by Sun et al. (2018) to address the occlusion in a complex background and to improve the organ detection (i.e., flower, fruit, stem, etc.) of tomatoes using a CNN. Based on the faster R-CNN, a network was developed using ResNet50 in the place of the VGG16 network. Finally, K-means clustering was used to enhance the detection accuracy by adjusting the anchor (i.e., bounding box) sizes. The developed system demonstrated improved detection accuracy when compared with traditional systems. Still, more works needs to be carried out in this context to achieve improved accuracy.

Overlapping and illumination variations, among other important issues, cause the poor detection of fruits in an orchard. To tackle these issues, a system was developed by Guo et al. (2019) where the background is first segmented using contrast limited adaptive histogram equalization combined with Otsu thresholding and morphological operations. Further, lychee fruit detection is performed from the overlapped fruits using the three-point definite circle theorem. Finally, a local binary pattern SVM is employed to reduce the false positive detections. However, more work is progressing in this area for improved detection accuracy.

#### Deep Learning Architecture

DL parameters greatly influence detection accuracy. When training DL systems, the primary learning parameters (i.e., weights and biases) need to be optimized for improved prediction (Koirala et al., 2019). In addition, hyper-parameters such as learning rate and momentum are adjusted to obtain the optimized architecture, so as to achieve superior results during the testing phase. It is important to note that the deeper layers extract more abstract features than the shallower networks. As a bonus, pre-trained networks with deeper layers provide superior results. However, these networks require a large memory and high amounts of computation time. When going deeper, the number of features extracted and the time needed to process the large volume of data are the main challenging issues in these architectures. Thus, a tradeoff is to be maintained between the accuracy and computational complexity in order to provide acceptable results from machine intelligence that matches human intelligence.

#### Computation Time

Computation time is an influential factor for any real-time system. Researchers have used different CPUs (Central Processing Units), GPUs (Graphics Processing Units), and image resolutions for fruit detection. The training time of any architecture depends on the batch size, the available memory, and the type of GPU. More importance has to be given to the testing time, as it operates on real-time data. Moreover, the complexity of an architecture will decide the computation speed of the network.

In a comparative study, done by Hani et al. (2019) showed that the different architectures count the fruits per image at different times. They used three architectures, namely, U-Net, the faster R-CNN, and the GMM. The input image patch of 224 × 224 pixel given to the U-Net takes less than 100 ms per image patch. The original input image of 1,920 × 1,080 pixel takes less than 4.5 s per frame. On the other hand, a faster R-CNN requires 120 ms per image patch (500 × 500) and 46 s per frame (1,920 × 1,080). A GMM runs at 5 frames per sec. These computations timings were obtained with the GPU of NVIDIA Quadro M1000 used in the proposed method. The video frames were obtained with 30 frames per sec and move at a speed of 2 m/s.

#### Confidence Score

The class probability score, called the confidence score, is used whereby values between 0 and 1 are assigned for object detection. Based on the detection of an object of a particular class, the values can be assigned. Generally, softmax probability is used in most of the detection tasks as a probability score. The threshold value for the probability score has to be fixed appropriately for improved pixel- based prediction. NMS is one of the thresholds used for detecting a single object by drawing bounding boxes. The greedy NMS algorithm is generally used for assigning windows. This method chooses the best scoring window and nominates a minimum value for suppressing the remaining windows after calculating the similarity between windows. The drawback is that sometimes it suppresses the window that allows for a superior choice for a particular object. It can be overcome by an alternative method that replaces the objective function as a cluster exemplar. Using that, all the similar windows are grouped and act as a single window for object detection (Rothe et al., 2015; Hosang et al., 2017).

#### Performance Evaluation

Various performance measures are used in semantic segmentation algorithms as described in section “Fully Convolutional Prediction.” Based on the exploration of these measures, the F1 score is considered optimum since it accounts for the harmonic mean of both precision and recall. Further, IoU allows for an improved estimation of overlapping and coincidence between the ground truth and the predicted pixels. Pixel accuracy provides poor results when there is a greater imbalance in the fruit or non-fruit classes (Yu et al., 2018; Hani et al., 2019; Kestur et al., 2019).

Bargoti and Underwood (2017) proposed a method for segmenting apples using two feature learning algorithms: multilayered perceptron and a CNN. The authors analyzed the architectures by adding metadata. The F1 score was used to measure the pixel-based prediction by comparing the two algorithms with and without metadata. The F1 score (0.839) was reasonably improved after adding the metadata in the multilayered perceptron.

Liu et al. (2019) developed a method for detecting kiwi fruit using image and feature fusion by capturing images in two different modalities (RGB and NIR). The average precision obtained by NIR (89.2%) was higher than the RGB (88.4%) since NIR images were less sensitive to image brightness changes occurring due to natural illumination. Hence, the performance metric results also depend on the way to capture the images. Image fusion method resulted in the best average precision (90.7%) among the all whereas the feature fusion gave the significantly closer result of 90.5%.

## Conclusion

This paper reviewed the various steps involved in intelligent fruit yield estimation, such as sampling, data collection, annotation and augmentation, fruit detection and counting, performance evaluation, and challenges using DL-based semantic segmentation architectures. The DL-based systems rectified the challenges of feature descriptors for object detection and hand-crafted feature learning methods. The stack of layers present in hierarchical learning improves the prediction accuracy of fruit detection at the cost of increased computational complexity. Transfer learning methods in DL and publicly available datasets are advantageous; optimized weights are used to train the architecture, and on-site fruit detection can be performed using these optimized networks. Accurate yield mapping for further harvesting and marketing can be performed smartly using these intelligent fruit yield estimation systems composed of DL-based semantic segmentation architectures.

However, the annotation of fruit images collected from the orchards is very tedious, time-consuming and needs improvement. The difficulty of detecting fruits in clustered and occluded regions need to be further explored using these architectures. Even though DL-based semantic segmentation architectures provide better results, a lightweight model is yet to be developed for smart-phone applications with less computational complexity. Future research can focus on the remedial measures for the issues of (manual) annotation, a comprehensive model for tackling the challenging conditions like occlusion, overlapping and illumination variation in the field, customization of lightweight model for android applications, etc.

## Author Contributions

PM and PR conceived and identified the outline. PM drafted the manuscript. OA-A provided suggestions and redesigned the figures. PR and MP-R performed the critical revision. All authors contributed to the article and approved the submitted version.

## Conflict of Interest

The authors declare that the research was conducted in the absence of any commercial or financial relationships that could be construed as a potential conflict of interest.

## Footnotes

1 https://org/project/labelImg/1.4.0/

2 https://labelbox.com

3 https://robots.ox.ac.uk/∼vgg/software/via/

4 https://appen.com

## Abbreviations

AI: Artificial Intelligence
AVIRIS: Airborne Visible/Infrared Imaging Spectrometer
CCD: Charge Coupled Device
CNN: Convolutional Neural Networks
CPU: Central Processing Unit
CART: Classification and Regression Trees Classifier
CMOS: Complementary Metal-Oxide-Semiconductor
CRAID: CRanberry Aerial Image Dataset
CRF: Conditional Random Fields
DL: Deep learning
ECa: apparent Electrical Conductivity
EM: Expectation-Maximization
FCN: Fully Convolutional Networks
GFPN: Gate Feature Pyramid Network
GMM: Gaussian Mixture Model
GPU: Graphics Processing Unit
HOG: Histograms of Oriented Gradient
IoU: Intersection over Union
LSTM: Long Short-Term Memory
MIL: Multiple Instance Learning
ML: Machine Learning
MRF: Markov Random Fields
NDVI: Normalized Difference Vegetation Index
NMS: Non-Maximum Suppression
PA: Precision Agriculture
R-CNN: Regions with Convolutional Neural Networks
ReLU: Rectified Linear Unit
ResNet: Residual Network
RMSE: Root Mean Squared Error
RPN: Region Proposal Network
SATD: Sum of Absolute Transformed Difference
SIFT: Scale Invariant Feature Transform
SUR: Systematic Uniform Random
SURF: Speeded-Up Robust Features
SVM: Support Vector Machines
UAS: Unmanned Aerial Systems
UAV: Unmanned Aerial Vehicle
UGV: Unmanned Ground Vehicle
VGG16: Visual Geometry Group16
VGG19: Visual Geometry Group19
YOLO: You Only Look Once
ZFNet: Zeiler and Fergus Network.
